# Pretreatment of Lignocellulosic Biomass with Cattle Rumen Fluid for Methane Production: Fate of Added Rumen Microbes and Indigenous Microbes of Methane Seed Sludge

**DOI:** 10.1264/jsme2.ME19113

**Published:** 2019-12-27

**Authors:** Yasunori Baba, Yu Matsuki, Shuhei Takizawa, Yoshihisa Suyama, Chika Tada, Yasuhiro Fukuda, Masanori Saito, Yutaka Nakai

**Affiliations:** 1 Laboratory of Sustainable Animal Environmental Science, Graduate School of Agricultural Science, Tohoku University Yomogida 232–3, Naruko-onsen, Osaki, Miyagi 989–6711 Japan; 2 Research Fellow of the Japanese Society for the Promotion of Science (JSPS) Japan; 3 Laboratory of Forest Ecology, Graduate School of Agricultural Science, Tohoku University Yomogida 232–3, Naruko-onsen, Osaki, Miyagi 989–6711 Japan; 4 Laboratory of Environmental Crop Science, Graduate School of Agricultural Science, Tohoku University Yomogida 232–3, Naruko-onsen, Osaki, Miyagi 989–6711 Japan; 5 Research Institute for Bioresources and Biotechnology, Ishikawa Prefectural University Suematsu1–308, Nonoichi, Ishikawa 921–8836 Japan; 6 Department of Agro-Food Science, Faculty of Agro-Food Science, Niigata Agro-Food University 2416 Hiranedai, Tainai, Niigata, 959–2702 Japan

**Keywords:** lignocellulose, rumen, methane fermentation, MiSeq, bioaugmentation

## Abstract

The pretreatment of lignocellulosic substrates with cattle rumen fluid was successfully developed to increase methane production. In the present study, a 16S rRNA gene-targeted amplicon sequencing approach using the MiSeq platform was applied to elucidate the effects of the rumen fluid treatment on the microbial community structure in laboratory-scale batch methane fermenters. Methane production in fermenters fed rumen fluid-treated rapeseed (2,077.3 mL CH_4_ reactor^−1^ for a 6-h treatment) was markedly higher than that in fermenters fed untreated rapeseed (1,325.8 mL CH_4_ reactor^−1^). Microbial community profiling showed that the relative abundance of known lignocellulose-degrading bacteria corresponded to lignocellulose-degrading enzymatic activities. Some dominant indigenous cellulolytic and hemicellulolytic bacteria in seed sludge (*e.g*., *Cellulosilyticum lentocellum* and *Ruminococcus flavefaciens*) and rumen fluid (*e.g*., *Butyrivibrio fibrisolvens* and *Prevotella ruminicola*) became undetectable or markedly decreased in abundance in the fermenters fed rumen fluid-treated rapeseed, whereas some bacteria derived from seed sludge (*e.g*., *Ruminofilibacter xylanolyticum*) and rumen fluid (*e.g*., *R. albus*) remained detectable until the completion of methane production. Thus, several lignocellulose-degrading bacteria associated with rumen fluid proliferated in the fermenters, and may play an important role in the degradation of lignocellulosic compounds in the fermenter.

Lignocellulosic biomass, composed of cellulose, hemicellulose, and lignin, is the most abundant natural polymeric carbon source in the world (17) and, thus, is an attractive candidate for use in methane production. However, due to the resistance of lignocellulose to fermentation, methane yield is generally low (36). Three types of biological pretreatment are currently being investigated in an attempt to increase methane production efficiency using lignocellulosic biomass: a pretreatment with enzymes, pure cultures, or mixed cultures (40). An enzymatic pretreatment is convenient because lignocellulolytic enzymes may be directly added to the reactor. However, due to the complications associated with lignocellulose, currently available enzymes alone do not effectively degrade lignocellulose; therefore, enzymes are always used with other pretreatment methods, such as an alkali pretreatment (40). A pretreatment using pure cultures, such as white-rot fungi, is a powerful method because these fungi produce many types of lignocellulolytic enzymes that favorably enhance biogas and bioethanol production (1, 3, 4, 43). However, this method is prolonged (several weeks) and requires sterilization. In contrast, the mixed-culture pretreatment (*e.g*., with rumen fluid, the liquid fraction of digestate from anaerobic digesters) results in a process that is rapid (several d) and does not require sterilization. We previously investigated the preservation of cattle rumen fluid for application to methane production (38) (Nakai, Y. *et al*. 2017, Patent No. US 9574213 B2) and its potential for the pretreatment of lignocellulosic substrates, and the findings obtained revealed that the rumen fluid treatment improved the degradation rates of cellulose, hemicellulose, and lignin and achieved 1.5–3.2-fold greater methane production than non-treatment systems (5, 7, 37). However, the (i) fate of the added rumen microbial community during methane production and (ii) the impact of the addition of such a different microbial community on the indigenous bacteria of methane seed sludge currently remain unclear. If the added rumen microbial community survives and continues to contribute to methane fermenter efficiency, it may be possible to utilize rumen microbes for the bioaugmentation of methane fermenters (*i.e*., one-phase biogas digesters). However, if added rumen microbes rapidly die out within the methane fermenter, they are applicable during only the first (hydrolytic) phase in two-phase (hydrolytic phase+methanogenic phase) biogas digesters.

The present study focused on lignocellulose-degrading bacteria and methanogenic archaea that contribute to the methanogenesis of lignocellulose and investigated the impact of adding rumen fluid containing an endogenous microbial community on the indigenous bacteria of methane seed sludge as well as the fate of the added rumen microbial community via MiSeq next-generation sequencing during methane fermentation. Previous studies (13, 24, 26, 31, 34, 41) reported the addition of lignocellulolytic pure cultures to methane fermenters; however, only a few (33, 44) have described the addition of entire rumen microbial communities or investigated their community structure. Furthermore, these studies were snapshots (*i.e*., microbial community structures at a specific time) of methane fermenters fed rumen fluid. The present study monitored time-dependent changes in the microbial community structure and investigated the fate of rumen-derived lignocellulose-degrading bacteria and indigenous lignocellulose-degrading bacteria of methane seed sludge during methane fermentation.

## Materials and Methods

### Feedstock

The stems and leaves of rapeseed (*Brassica napus*) were air-dried and milled to produce lignocellulosic biomass that was used as the substrate for methane production after the pretreatment with rumen fluid.

### Rumen fluid treatment

Rumen contents were orally harvested from cattle, filtered through a mesh strainer (1×1 mm pores) to remove coarse solids, and stored at 37°C. Prepared rapeseed (9.0 g dry weight) was pretreated with 300 mL of rumen fluid at 37°C on a rotary shaker at 170 rpm for 6 or 24 h. The rumen fluid treatment was performed in duplicate. The care and use of animals in the present study were approved by the Institutional Animal Care and Use Committee of Tohoku University.

### Methane production

Methane seed sludge for the initiation of the experiment was prepared using anaerobically digested sludge collected from the biogas plant at Tohoku University (Osaki, Japan). Some substrates (6) were then added to the sludge, which was used as the sludge for the start-up of the experiment. The experiment commenced when the sludge stopped biological activity, as assessed by the cessation of biogas production. Batch-type methane production was performed in 1,000-mL reactors (600-mL working volume: 200 mL of pretreated and untreated rapeseed was added to 400 mL of seed sludge) and incubated at 35°C for 32 d. Deionized water was used instead of rumen fluid in the control. The methane production process and 16S rRNA sequencing were performed in singlicate.

### Analysis

The biomass chemical composition was analyzed by the Agricultural Product Chemical Research Laboratory in the Tokachi Federation of Agricultural Cooperatives using the detergent fiber method (39) and estimation equations of the National Research Council (NRC) (28). Total solids (TS) and volatile solids (VS) were assessed using standard methods (2). Biogas (CH_4_, CO_2_, and H_2_) concentrations were evaluated using a gas chromatograph (GC-8A; Shimadzu, Kyoto, Japan), volatile fatty acid (VFA) concentrations were measured by a high-performance liquid chromatograph (JASCO, Tokyo, Japan), and chemical oxygen demand (COD) was assessed using a colorimetric method based on Cr, as previously described (5). Lignocellulose (lignin, cellulose, xylan)-degrading enzyme activity was measured as previously described (7). DNA extraction was performed using a Power Soil DNA kit (MO BIO, Carlsbad, CA, USA), 16S rRNA gene MiSeq sequencing was performed using the FwOvAd_341F (forward) and ReOvAd_785R (reverse) primer set, and an analysis by QIIME software with a 97% threshold for the OTU definition was conducted as previously described (7). Taxonomic identification was performed at the phylum and genus levels. The representative (most abundant) sequence of each genus was investigated by the basic local alignment search tool (BLAST, http://blast.ncbi.nlm.nih.gov/Blast.cgi) to confirm the closest species. All sequence data were deposited in the DRA of the DDBJ database under DRA008816.

## Results and Discussion

### Feedstock and rumen fluid

The chemical composition of rapeseed was as follows: 39.4% non-fibrous carbohydrate, 21.7% cellulose, 3.3% hemicellulose, 3.2% lignin, and other components (such as protein and ash). The TS and VS compositions of rapeseed were 91.9 and 74.2%, respectively. The TS and VS of rumen fluid after filtration were 1.6 and 0.8%, respectively.

### Methane production from lignocellulose pretreated with rumen fluid

**Fig. 1:**
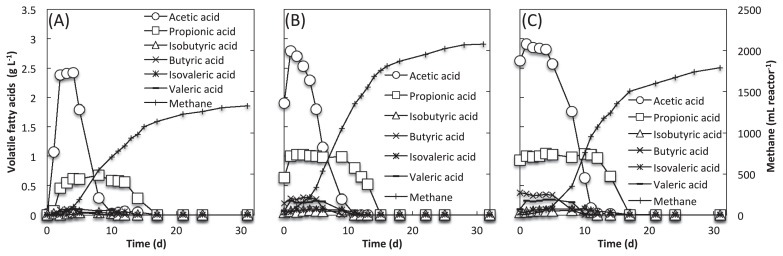
Time course of VFA and methane production in methane fermenters fed untreated rapeseed (A) and rapeseed pretreated with rumen fluid for 6 h (B) and 24 h (C).

Rapeseed was soaked with rumen fluid for 6 or 24 h prior to the initiation of methane production. Rapeseed was solubilized, resulting in the production of various VFAs, such as acetate and propionate, as metabolites of plant cell wall components (*e.g*., cellulose, hemicellulose, and lignin) (Table S1). Batch-type methane production was performed using pretreated and untreated (control) rapeseed. VFAs underwent conversion to methane over time (Fig. 1). More methane was produced from pretreated rapeseed (*i.e*., 2,077.3 mL CH_4_ reactor^−1^ from 6-h pretreated rapeseed; 1,790.4 mL CH_4_ reactor^−1^ from 24-h pretreated rapeseed) than from untreated control rapeseed (*i.e*., 1,325.8 mL CH_4_ reactor^−1^), which is consistent with previous findings (5).

### Sampling depth, coverage, and taxonomic composition of the microbial community during the methane production process

After quality filtering, paired-end read assembly, quality control, and chimera removal, 2,663,094 V3–V4 16S rRNA sequence reads (average 126,814; minimum 46,723; maximum 197,504) were obtained from raw MiSeq data from 21 samples. The average sequence read length after primer subtraction was 450.9 bp. With a subsample of 46,723 reads per sample, rarefaction curves (Fig. S1) demonstrated that most of the authors’ sampling efforts provided sufficient species coverage to accurately describe the bacterial composition of each sample.

**Fig. 2:**
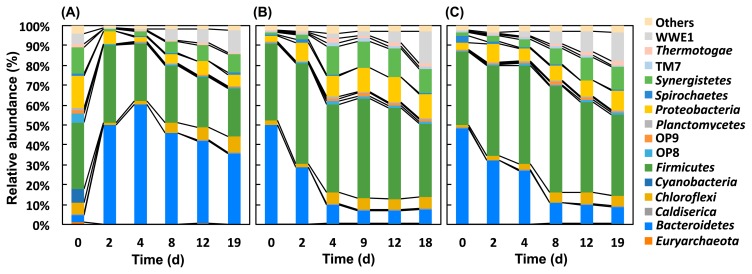
Changes in the phylum-level taxonomic composition of methane fermenters fed untreated rapeseed (A) and rapeseed pretreated with rumen fluid for 6 h (B) and 24 h (C). Fifty-two phyla were identified. The abundance of 37 of these was <1%, and, thus, these phyla were included in the “Others” category.

Fifty-one phyla were identified within the microbial community structure during the methane production process. The abundance of 36 of these phyla was <1.0%, and these were included in the “Others” category (Fig. 2). The relative abundance of *Synergistetes* and WWE1 increased over time. *Aminobacterium* was the main member of the phylum *Synergistetes* in the methane fermenter fed untreated rapeseed, whereas *Thermovirgaceae*, vadinCA02, HA73, and *Aminobacterium* were the main members in the fermenter fed with rumen fluid-pretreated rapeseed (Fig. S2). The most abundant sequences of *Aminobacterium*, *Thermovirgaceae*, and HA73 were associated with known amino acid-degrading bacteria (*i.e*., members of *Aminobacterium* [8], *Thermovirga* [16], and *Aminivibrio* [19]) (Table S2). The most abundant sequence of vadinCA02 was associated with unknown bacteria. W22 and W5 were detected in the phylum WWE1, and the most abundant sequences were associated with unknown bacteria (Fig. S3 and Table S3). The phylum *Bacteroidetes* exhibited the most significant changes. Taxa with relative abundance >1.0% that belong to *Bacteroidetes* are shown in Fig. S4. In the untreated control, *Bacteroides*, *Porphyromonadaceae*, and *Prevotella* were the main members of *Bacteroidetes*, and their relative abundance increased after the addition of rapeseed. The most abundant gene sequences of these taxa were associated with known plant cell component (*i.e*., xylan, cellobiose, starch, and protein)-degrading bacteria (*i.e*., members of *Bacteroides* [29], *Proteiniphilum* [14], and *Prevotella* [25]) (Table S4), and, thus, the increases observed in these bacteria were reasonable. In rumen fluid-pretreated rapeseed, the phylum *Bacteroidetes* was the most abundant on day 0 because many *Bacteroidetes* migrated from the pretreatment reactor to the methane fermenter (Fig. 2). *Prevotella*—originating from pretreatment fluid (Fig. S4)—was detected as the main member of *Bacteroidetes* on day 0; however, the relative abundance of this genus decreased over time. Similarly, the total number of observed OTUs in fermenters with mixed pretreatment fluids decreased with time to the same level as in the original seed sludge (Fig. S5). Collectively, these results suggest that many rumen-derived microbes may not survive in the methane fermenter.

**Fig. 3:**
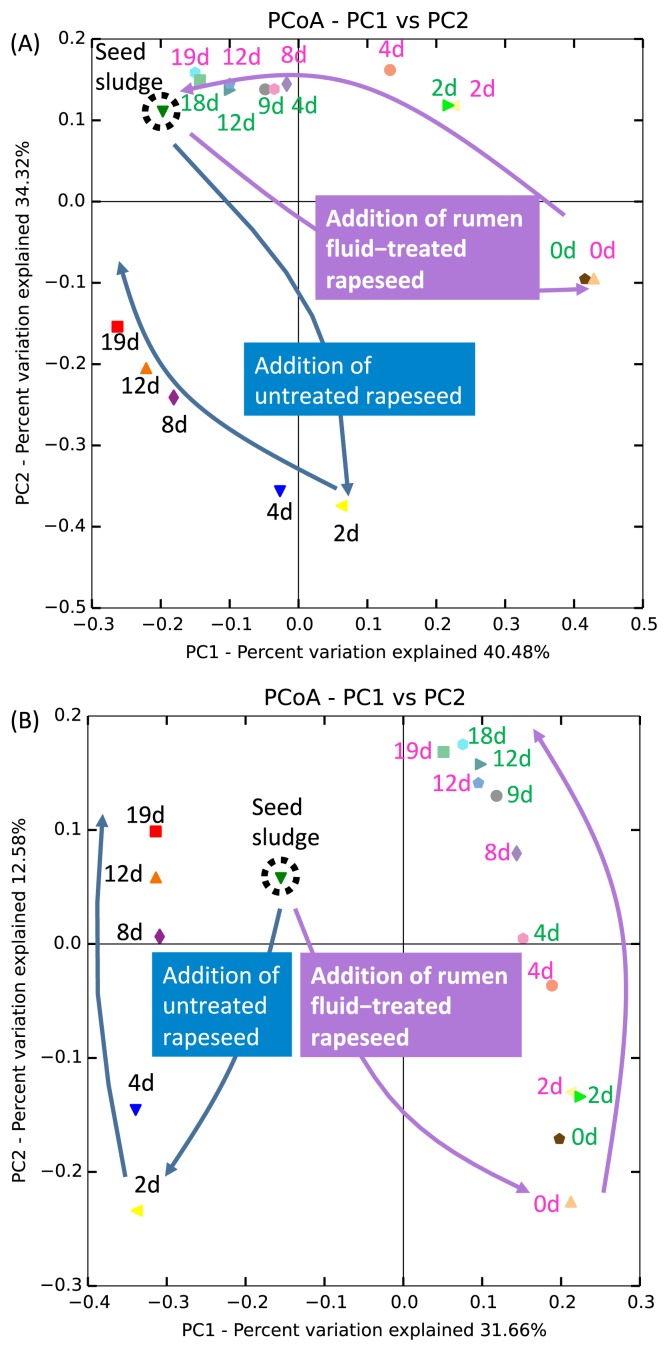
Principal coordinate analysis (PCoA) of weighted (A) and unweighted (B) UniFrac distances of microbial 16S rRNA sequences (sourced from methane fermentation samples) from the V3–V4 region. d, days. Colors of time points: black=control (*i.e*., methane fermenter fed untreated rapeseed), green=methane fermenter fed rapeseed pretreated with rumen fluid for 6 h, and pink=methane fermenter fed rapeseed pretreated with rumen fluid for 24 h.

To elucidate community structure alterations caused by the addition of pretreatment fluid, a weighted principal coordinate analysis (PCoA) (*i.e*., consideration of the relative abundance of each OTU) was performed (Fig. 3A). The results obtained indicated that the structure of the microbial community in the methane fermenter on day 0—immediately after mixing with pretreatment fluid—markedly differed from that of the seed sludge. The microbial community structure of the fermenter contents mixed with pretreatment fluid then roughly returned to that of seed sludge. On the other hand, an unweighted PCoA (Fig. 3B) was performed to elucidate differences in community members (*i.e*., not considering the relative abundance of each OTU), and the results obtained indicated that the composition in the fermenter mixed with pretreatment fluid did not return to that in the seed sludge. Alpha diversity indices (Shannon, ACE, and Chao1) also indicated that species richness increased due to the addition of pretreatment fluids (Table S5). Thus, these results indicated that although the addition of rumen fluid affected both the relative abundance and composition of the microbial communities of methane seed sludge, its addition had a stronger impact on the compositions of these communities.

### Lignocellulose-degrading bacteria indigenous to methane seed sludge

To estimate the composition of lignocellulose-degrading bacteria originating from methane seed sludge, the species most closely related to the most abundant sequence of each assigned taxon for methane fermentation without the pretreatment (*i.e*., control) was identified by a homology search of the GenBank database using BLAST. Nine OTUs that are phylogenetically related to known cellulolytic, xylanolytic, and aromatic-degrading bacteria were detected in the methane fermenter without the pretreatment (Table 1). Changes in the relative abundance of each bacterium and its lignocellulose-degrading enzyme activity are shown in Fig. S6. Regarding the cellulose-degrading population, changes in the relative abundance of the two OTUs 14124 and 342612, associated with known cellulolytic bacteria (*i.e*., members of *Cellulosilyticum* [12] and *Ruminococcus* [21]), mirrored the changes in cellulase (CMCase, Avicelase, and *β*-glucosidase) activities. Regarding the xylan-degrading population, changes in the relative abundance of OTU 572010, associated with known xylanolytic bacteria (*i.e*., members of *Bacteroides* [29]), mirrored the changes in cell-bound xylanase activities, whereas changes in the 4 other OTUs (544105, 768947, 14124, and 342612) associated with known xylanolytic bacteria (*i.e*., members of *Ruminofilibacter* [30], *Prevotella* [25], *Cellulosilyticum* [12], and *Ruminococcus* [21]) mirrored changes in free xylanase activities. Regarding the aromatic-degrading population, changes in the relative abundance of OTU 625556 mirrored those in manganese peroxidase (MnP) activity. This OTU was related to the aromatic-degrading bacteria *Syntrophorhabdus* (35); however, sequence homology was low (94%); therefore, OTU 625556 may be a new bacterium related to lignin degradation. The other 3 OTUs (659146, 840291, and 4403651) associated with known aromatic-degrading bacteria (*i.e*., members of *Sedimentibacter* [42], *Acinetobacter* [27], and *Pseudomonas* [11]) (Fig. S7) did not mirror the dynamics of any lignin-degrading enzyme activities. Changes in the relative abundance of most lignocellulose-degrading bacteria detected during the present study did not contradict those observed in the activities of the relevant enzymes. Therefore, these bacteria may play a direct role in lignocellulose degradation during methane fermentation.

### Impact of the addition of pretreatment fluid on bacteria indigenous to methane seed sludge

**Fig. 4:**
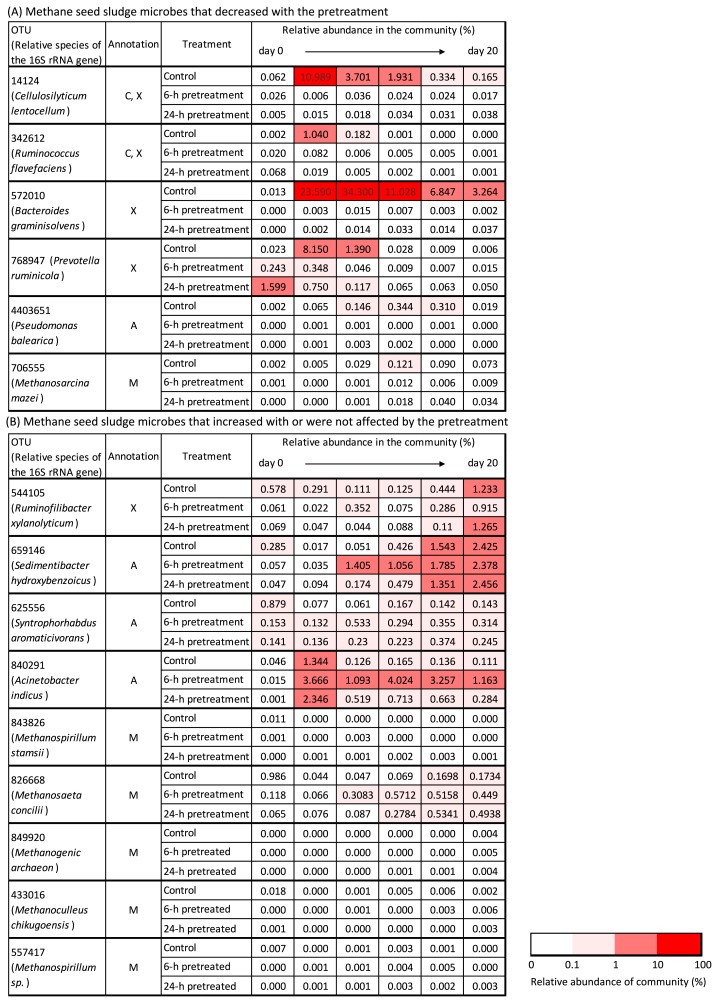
Impact of the addition of rapeseed pretreated with rumen fluid on the relative abundance of indigenous lignocellulose-degrading bacteria and methanogenic archaea in methane seed sludge. Annotation: C, cellulose-degrading bacteria; X, xylan-degrading bacteria; A, aromatic-degrading bacteria; M, methanogenic archaea.

The pretreatment fluid (*i.e*., rapeseed stem dissolved in rumen fluid) was added to the methane fermenter, and the impact of the addition of this fluid on the above-mentioned indigenous lignocellulose-degrading bacteria of methane seed sludge (Table 1) was investigated. The results obtained for methanogenic archaea (Table S6) were added to these results, and a summary of the results combined are shown in Fig. 4. Six out of 15 OTUs (14124, 342612, 572010, 768947, 4403651, and 706555) no longer increased in relative abundance with the addition of the pretreatment fluid; however, these OTUs increased in the methane fermenter fed untreated rapeseed (*i.e*., control). OTU14124, 342612, 572010, and 768947 were associated with known cellulolytic and xylanolytic bacteria (*i.e*., members of *Cellulosilyticum* [12], *Ruminococcus* [21], *Bacteroides* [29], and *Prevotella* [25]) and decreased by one to four orders of magnitude with the addition of the pretreatment fluid. The pretreatment fluid contained cellulose and hemicellulose (Table S1) as carbon and energy sources, and pH values (Fig. S8) during the methane fermentation period were within the physiological range of these bacteria, which may have grown in the reactor; however, their abundance did not increase. On the other hand, OTU 544105, associated with known xylanolytic bacteria (*i.e*., members of *Ruminofilibacter* [30]), increased in abundance, similar to that in the control. Regarding OTUs associated with known aromatic-degrading bacteria, OTU4403651 (*i.e*., members of *Pseudomonas* [11]) became undetectable with the addition of the pretreatment fluid at the end, whereas the other 3 OTUs (*i.e*., members of *Sedimentibacter* [42], *Syntrophohabdus* [35], and *Acinetobacter* [27]) in this category remained detectable. Regarding OTUs associated with known methanogenic archaea, the relative abundance of OTU706555 (*i.e*., a member of *Methanosarcina* [10]) decreased by one order of magnitude with the addition of the pretreatment fluid, whereas the other 5 OTUs (*i.e*., members of *Methanospirillum*, *Methanosaeta*, and *Methanoculleus*) (10) were not notably affected by the addition of the pretreatment fluid. In summary, the addition of the pretreatment fluid had a large impact on the fate of the indigenous cellulolytic and xylanolytic bacteria of methane seed sludge.

### Fate of added rumen microbes

**Fig. 5:**
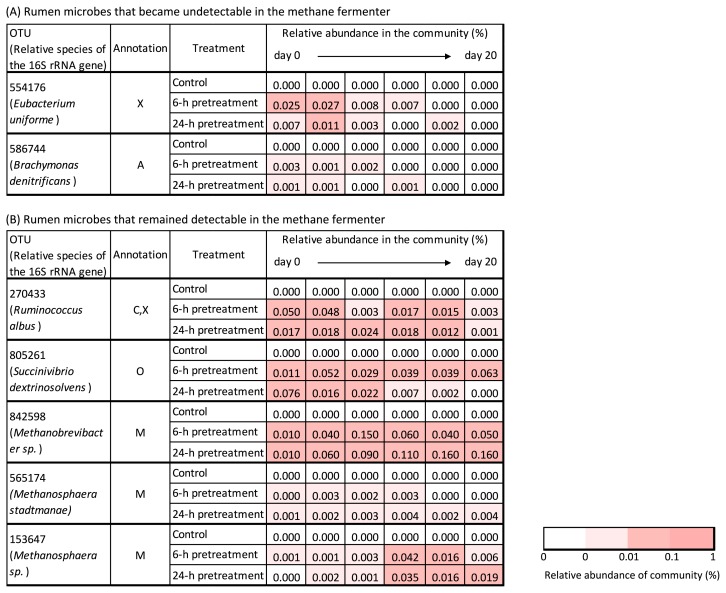
Changes in the relative abundance of lignocellulose-degrading bacteria and methanogenic archaea endemic to rumen fluid during the methane production process. Annotation: C, cellulose-degrading bacteria; X, xylan-degrading bacteria; A, aromatic-degrading bacteria; O, cello-oligosaccharide-degrading bacteria; M, methanogenic archaea.

The OTUs associated with known lignocellulose-degrading bacteria and methanogenic archaea detected in the rumen fluid treatment are shown in Table S7. The OTUs endemic to rumen fluid (*i.e*., not detected in methane seed sludge) are shown in Table S7, and changes in the relative abundance of the added rumen microbes during the methane production process are shown in Fig. 5. Two OTUs (554176, 586744) gradually became undetectable, whereas 5 OTUs (270433, 805261, 842598, 565174, and 153647) remained detectable until the completion of methane production. OTU 270433 was associated with a known cellulolytic rumen bacterium (*i.e*., *Ruminococcus albus* [25]). Sequences other than the above-mentioned most abundant sequences were also investigated among OTUs assigned as *Ruminococcus*, and 4 OTUs were sequences associated with *R. albus* and remained detectable until the completion of methane production (Fig. S9). Although the present study confirmed the existence of a 16S rRNA gene associated with *R. albus*, if *R. albus* is confirmed to actually degrade cellulose in methane fermenters, bioaugmentation using *R. albus* will be an effective means for methane production from lignocellulose. OTU 805261 was associated with a known cello-oligosaccharolytic rumen bacterium (*i.e*., a member of *Succinivibrio* [11]). OTU842598, 565174, and 153647 were associated with known H_2_-utilizing methanogenic archaea (*i.e*., members of *Methanobrevibacter* or *Methanosphaera*). These methanogenic archaea obtain energy through the reduction of methanol or CO_2_ to CH_4_ using H_2_ as a substrate (10). To achieve the successful degradation of aromatic compounds derived from lignin, the presence of H_2_-utilizing methanogens (which lower the H_2_ partial pressure in methane fermenters) is generally required (22, 35). Furthermore, one key reason for the collapse of methane fermentation is propionate accumulation (6, 18). To facilitate propionate degradation, propionate-oxidizing bacteria must thermodynamically coexist with H_2_-utilizing methanogens (20). The relative abundance of the two OTUs 591709 and 1129757, associated with known propionate-oxidizing bacteria (*i.e*., members of *Syntrophobacter* [9, 15]), slightly increased with the addition of the pretreatment fluid (Fig. S10 and Table S8). Therefore, the increase observed in the growth of the above-mentioned H_2_-utilizing methanogens derived from the pretreatment fluid may promote the continuation of robust methane fermentation.

Some of the OTUs (571178, 99264, 326292, and 589852) in Table S7 associated with known cellulolytic or xylanolytic rumen bacteria (*i.e*., members of *Butyrivibrio* [25], *Prevotella* [25, 33], and *Pseudobutyrivibrio* [23]), which were also detected in the control, are shown in Fig. S11. These OTUs migrated from the rumen pretreatment reactor to the methane fermenter and were dominant on day 0 in the pretreated methane fermenter. However, their abundance gradually decreased, resulting in the same levels as these bacteria detected in the control at the end of the experiment. Each bacterium of the complex microbial community may have a suitable existence ratio depending on their role.

The present study demonstrated that rumen-derived bacteria associated with known cellulolytic and hemicellulolytic bacteria (*i.e*., *R. albus*) remained detectable until the completion of the methane production process. Conversely, indigenous bacteria associated with known cellulolytic and hemicellulolytic bacteria (*e.g*., *Cellulosilyticum lentocellum*, *Bacteroides graminisolvens*, and *Prevotella ruminicola*) in methane seed sludge markedly decreased after the addition of the pretreatment fluid. In addition, a rumen-derived archaeon associated with known H_2_-utilizing methanogenic archaea (*i.e*., *Methanobrevibacter* sp. and *Methanosphaera* sp.) also remained detectable until the completion of the methane production process after the addition of pretreatment fluid. Thus, the methane fermenter in the present study became a unique fermenter composed of pretreatment fluid-derived cellulolytic, hemicellulolytic, and methanogenic rumen microbes and indigenous species in methane seed sludge. Differences in the species composition of the methane fermenter suggested by PCoA may be caused by these rumen microbes that survived. If these pretreatment fluid-derived rumen microbes persist in the methane fermenter, they are expected to enhance lignocellulose-based methane productivity. Future studies are needed to elucidate whether these microbes directly contribute to the enhanced efficiency of methane fermentation by confirming the presence of enzyme mRNA (*e.g*., that of cellulase or hemicellulase) in pretreatment fluid-derived rumen microbes within the methane fermenter.

## Conclusion

The rumen fluid pretreatment enhanced methane productivity. The microbial community structure analysis demonstrated that dominant indigenous cellulolytic and hemicellulolytic bacteria in methane seed sludge no longer increased or markedly decreased in relative abundance after the addition of the pretreatment fluid. Conversely, certain rumen-derived cellulolytic, hemicellulolytic, and H_2_-utilizing methanogenic microbes introduced into the fermenter via the pretreatment fluid inoculation remained detectable until the completion of methane production. Thus, these results showed that the methane fermenter in the present study is a unique fermenter composed of pretreatment fluid-derived rumen microbes and indigenous species in methane seed sludge and also that this microbial community structure may contribute to methane productivity.

## SUPPLEMENTARY MATERIAL



## Figures and Tables

**Table 1:** OTUs related to lignocellulose-degrading bacteria in methane seed sludge.

OTU ID	Relative species of 16S rRNA gene	Substrate	Accession no.	Identity
14124	*Cellulosilyticum lentocellum* DSM 5427	Cellulose, Xylan	NR_074536	441/442 (99%)
342612	*Ruminococcus flavefaciens* FD-1	Cellulose, Xylan	AM920691	433/440 (98%)
572010	*Bacteroides graminisolvens* XDT-1	Xylan	NR_041642	459/460 (99%)
544105	*Ruminofilibacter xylanolyticum* S1	Xylan	DQ141183	458/460 (99%)
768947	*Prevotella ruminicola* CG41	Xylan	AB849451	459/460 (99%)
659146	*Sedimentibacter hydroxybenzoicus* JW/Z-1	Aromatics	NR_029146	421/442 (95%)
625556	*Syntrophorhabdus aromaticivorans* UI	Aromatics	NR_041306	440/469 (94%)
840291	*Acinetobacter indicus* strain A648	Aromatics	NR_117784	465/466 (99%)
4403651	*Pseudomonas balearica* VITPS19	Aromatics	MF164145	464/465 (99%)

OTUs marked with gray color were not detected in rumen fluid but were endemic to methane seed sludge.
