# Supplementary material for: Pretreatment of Lignocellulosic Biomass with Cattle Rumen Fluid for Methane Production: Fate of Added Rumen Microbes and Indigenous Microbes of Methane Seed Sludge
Source: Microbes Environ. 2019 Dec 27;34(4):421–8. doi: 10.1264/jsme2.ME19113 (PMC6934390; doi:10.1264/jsme2.ME19113)
Supplement: Supplementary file 1 [file 34_421_s1.pdf]

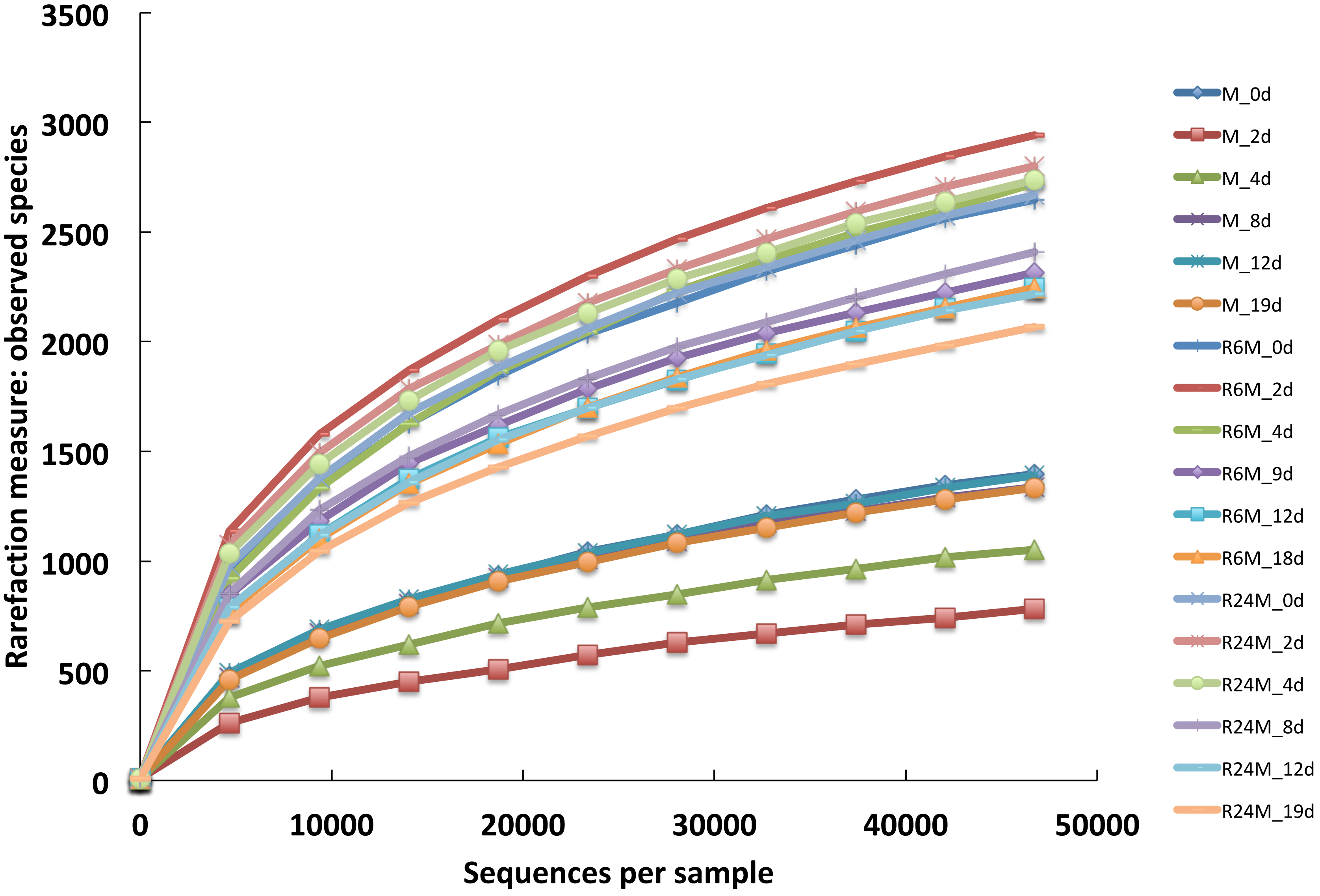

Fig. S1 (supplemental material)
Rarefaction curve of the bacteria in the respective samples.

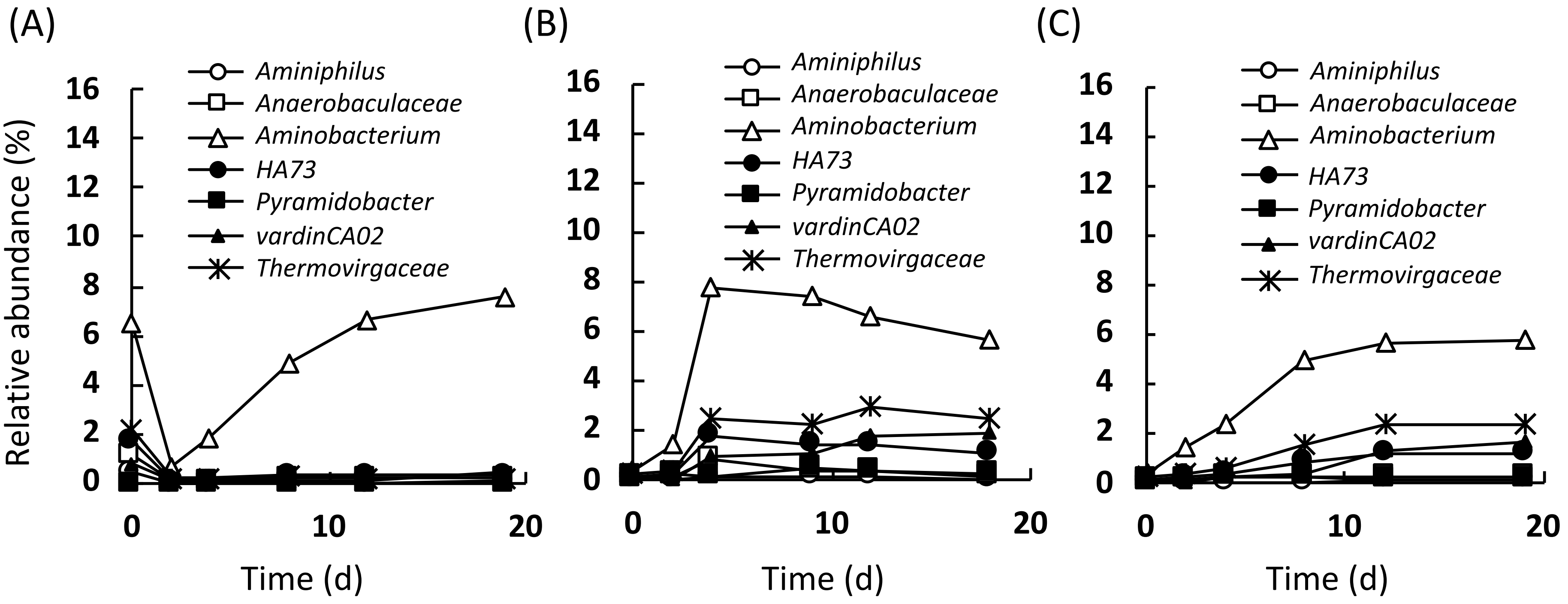

Fig. S2 (supplemental material)
Synergistetes taxa during methane fermentation fed untreated rapeseed (A) and rapeseed pretreated with rumen fluid for 6 h (B) and 24 h (C).

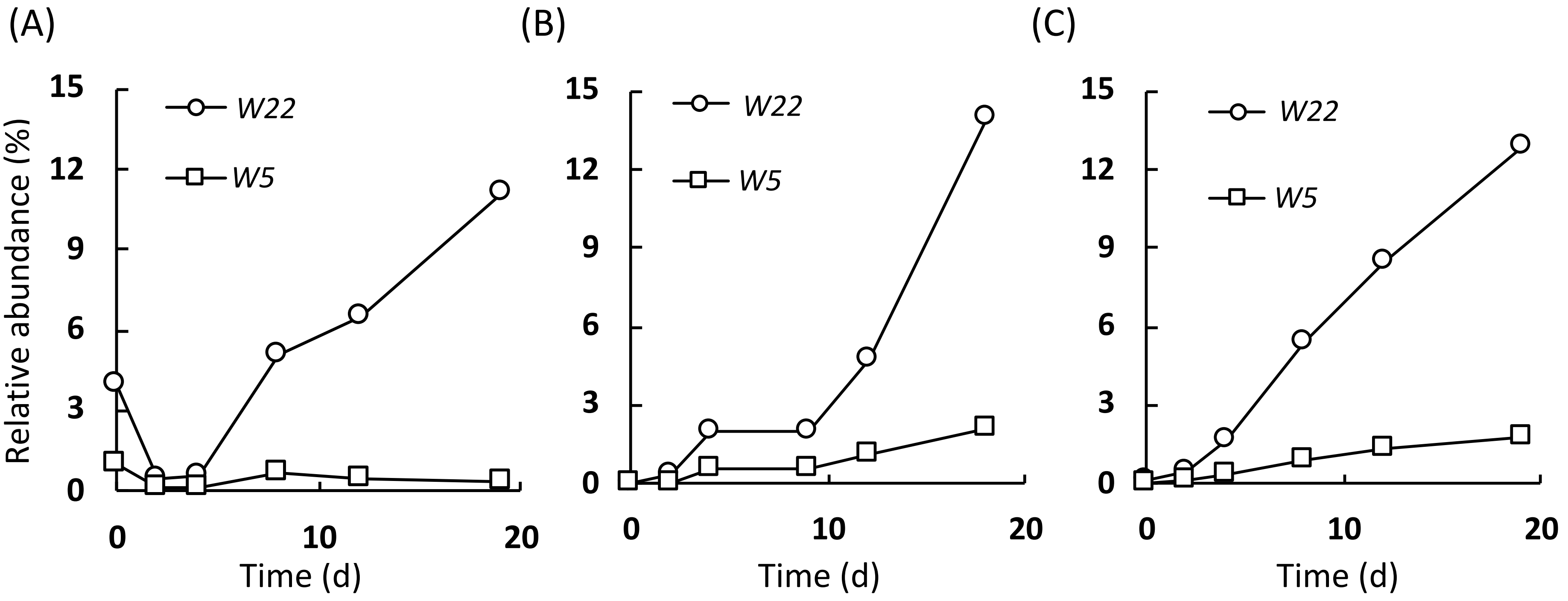

Fig. S3 (supplemental material)
WWE1 taxa during methane fermentation fed untreated rapeseed (A) and rapeseed pretreated with rumen fluid for 6 h (B) and 24 h (C).

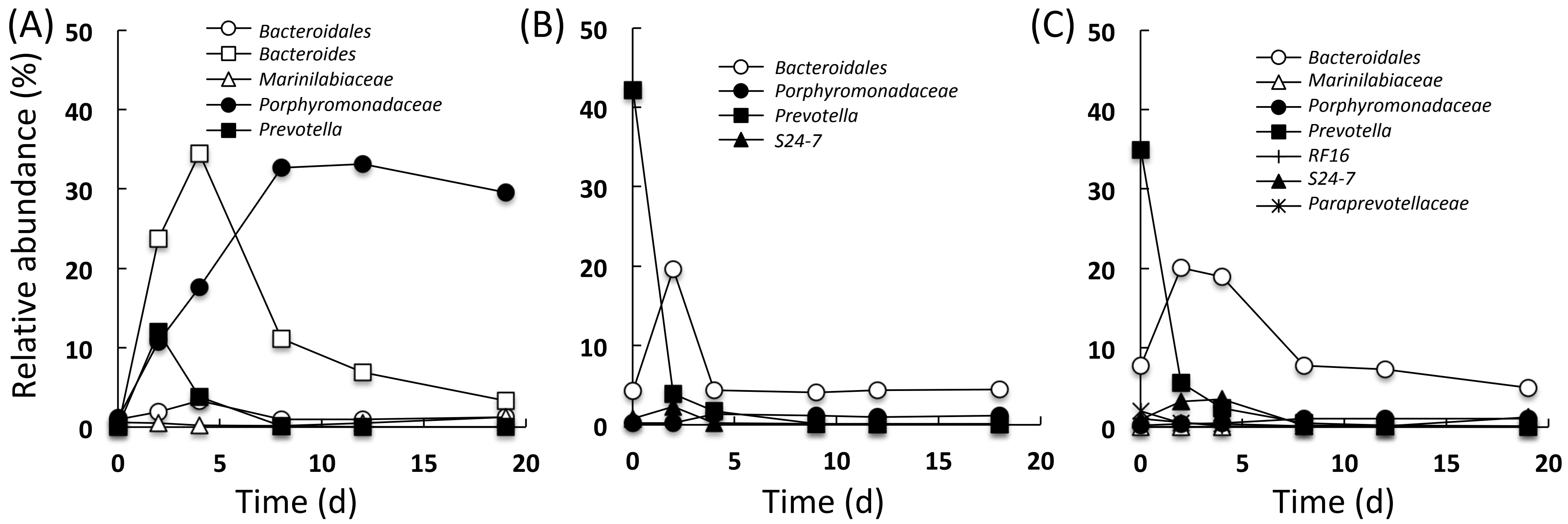

Fig. S4 (supplemental material)

Bacteroidetes taxa (>1.0%) during methane fermentation fed untreated rapeseed (A) and rapeseed pretreated with rumen fluid for 6 h (B) and 24 h (C).

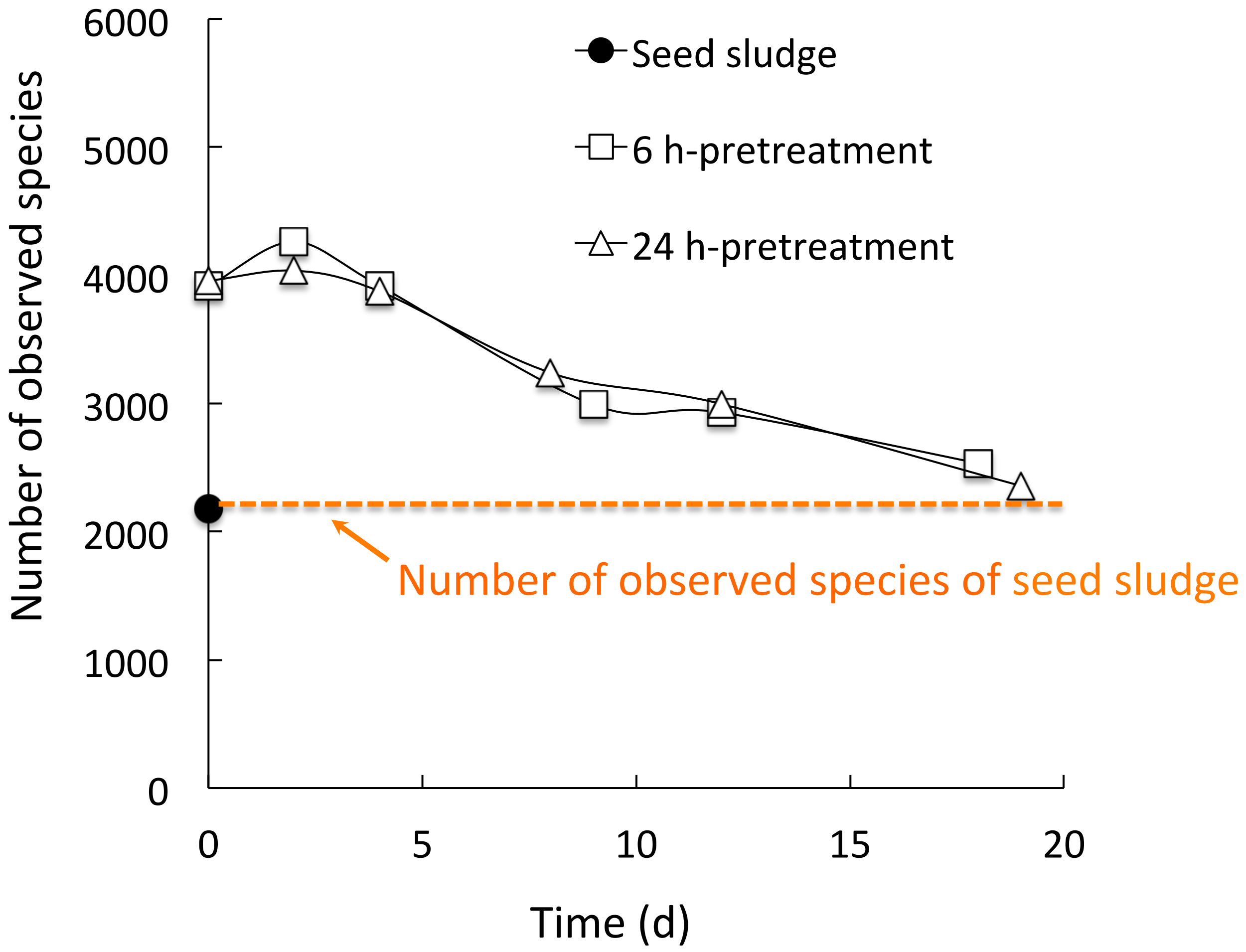

Fig. S5 (supplemental material)
Change in the number of species observed during methane fermentation after addition of the pretreatment fluid.

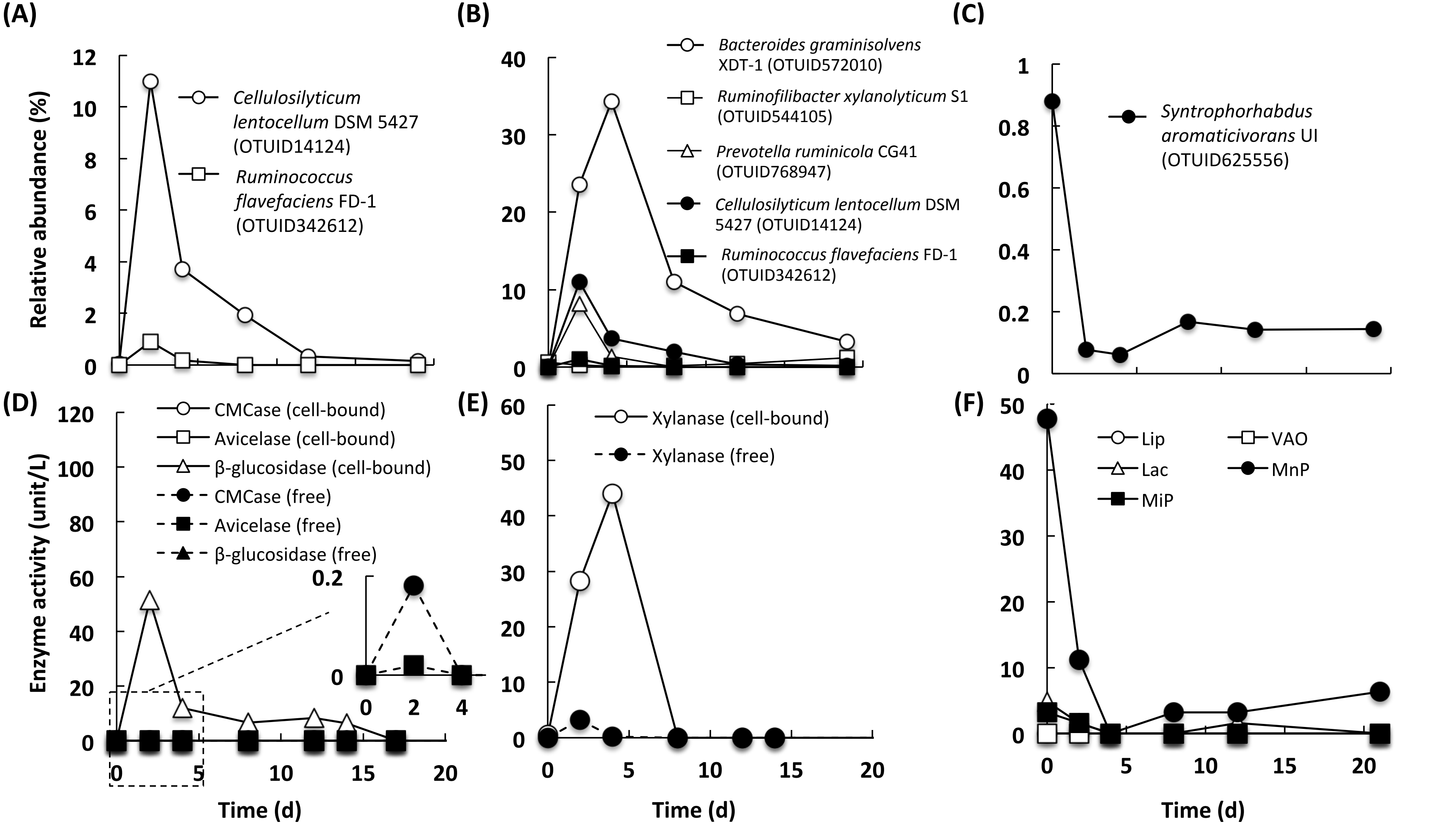

Fig. S6 (supplemental material)

Changes in the relative abundance of OTUs associated with known cellulose-degrading bacteria (A), xylan-degrading bacteria (B), or aromatic-degrading bacteria (C), and cellulase (D), xylanase (E), and ligninolytic enzyme (lignin peroxidase (Lip), veratryl alcohol oxidase (VAO), laccase (Lac), manganese peroxidase (MnP) and manganese-independent peroxidase (MiP)) (F) enzyme activities during methane fermentation of rapeseed. Subfigure (C) reports a subset of all detected aromatic-degrading bacteria.

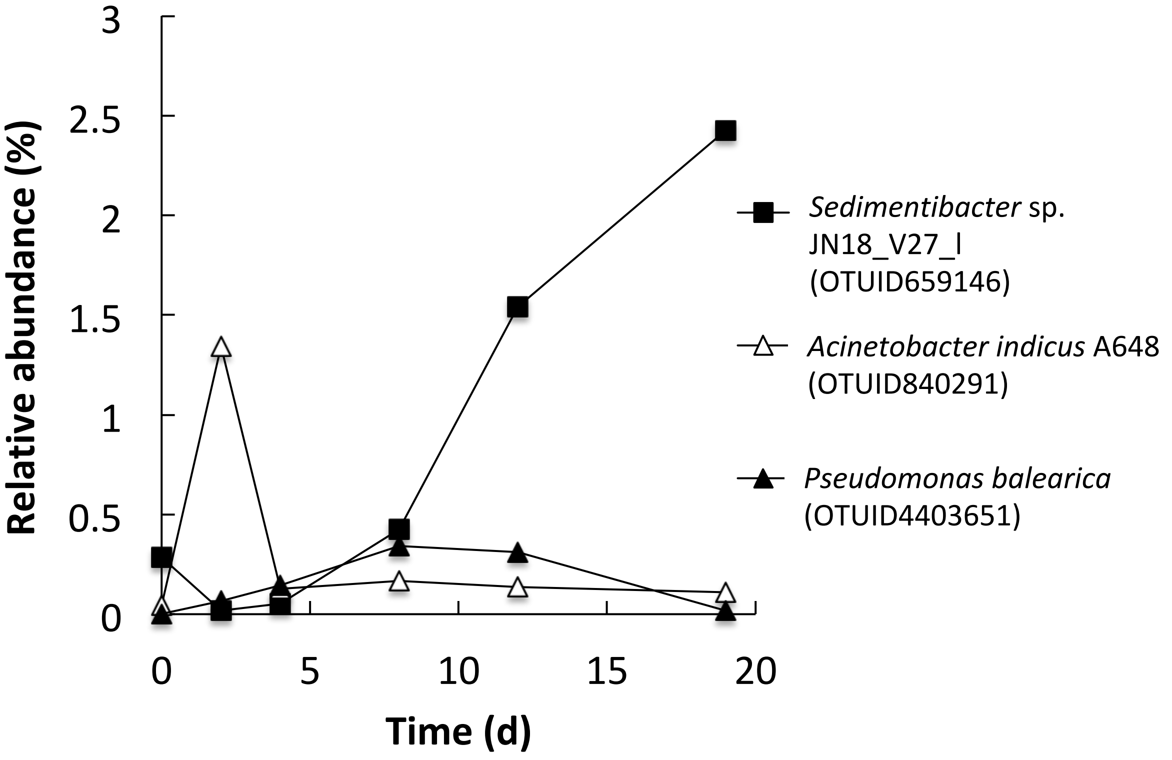

Fig. S7 (supplemental material)
Relative abundance of OTUs associated with known aromatic-degrading bacteria that did not mirror the dynamics of any lignin-degrading enzyme activities.

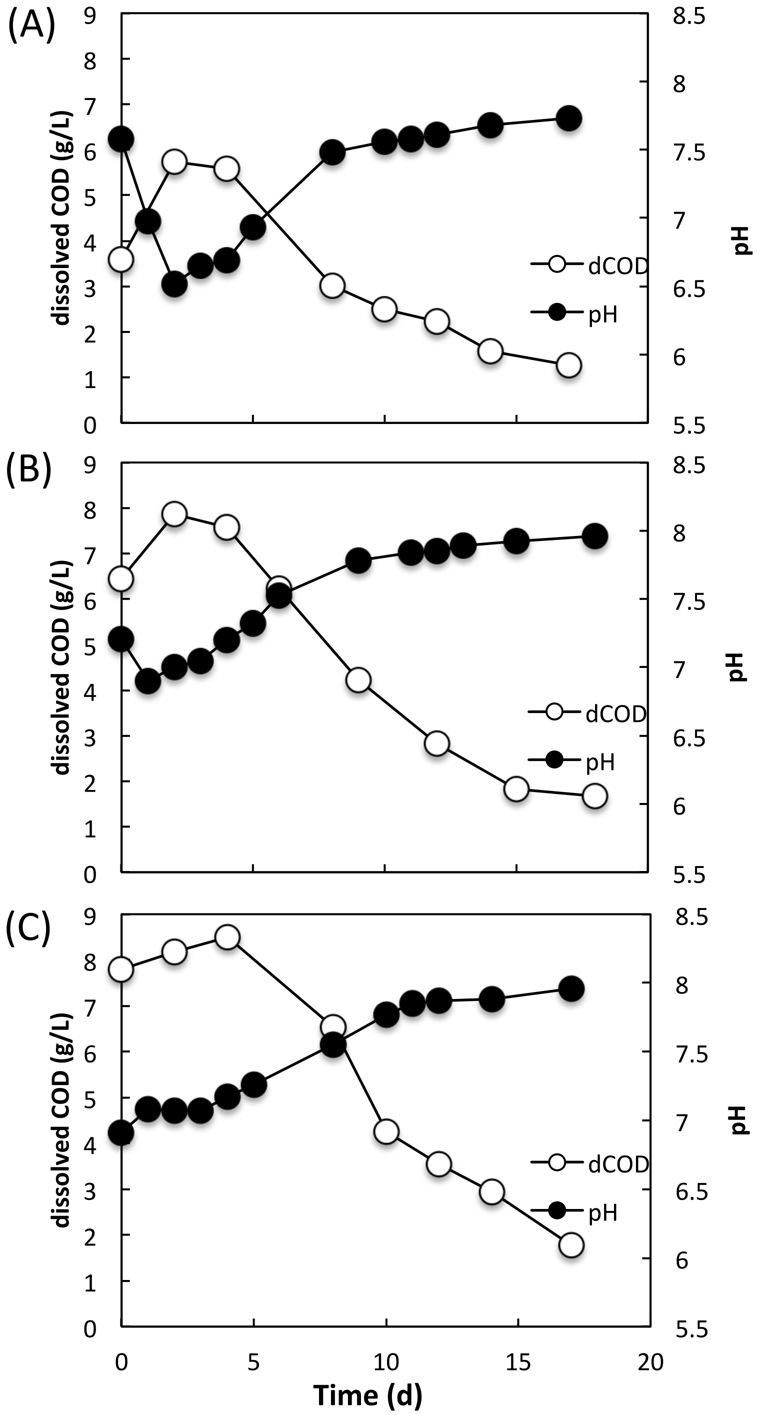

Fig. S8 (supplemental material)
Time course of dissolved COD and pH during methane fermentation fed untreated rapeseed (A) and rapeseed pretreated with rumen fluid for 6 h (B) and 24 h (C).

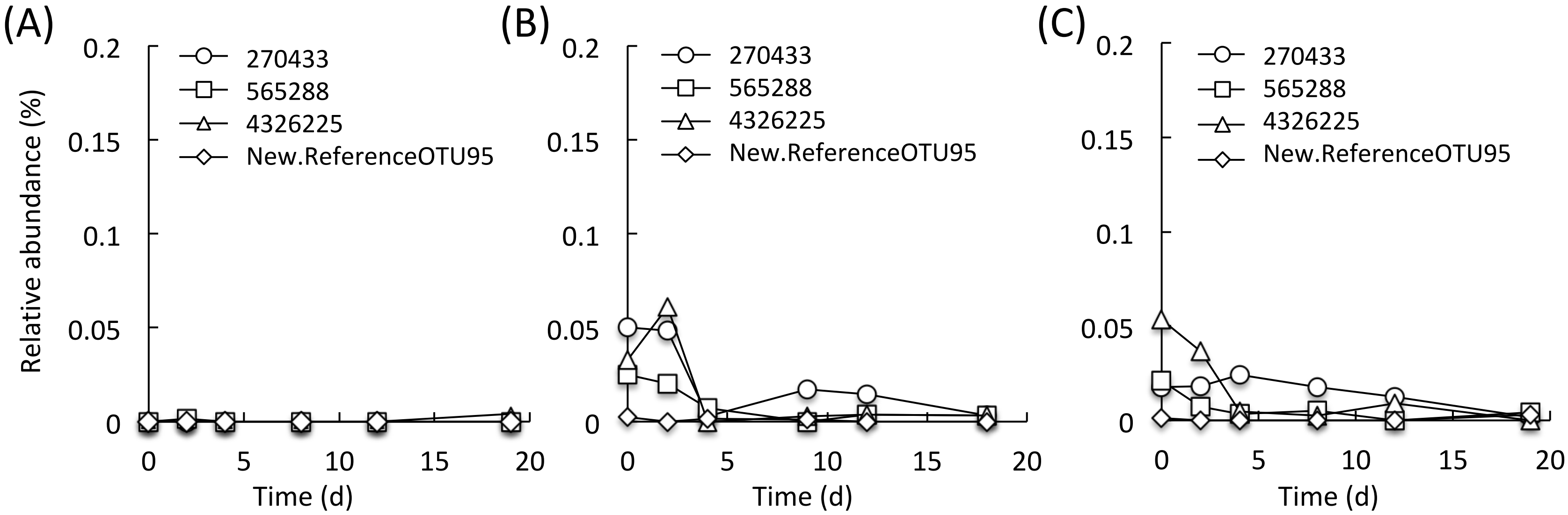

Fig. S9 (supplemental material)
Relative abundance of OTUs associated with *Ruminococcus albus* during methane fermentation fed untreated rapeseed (A) and rapeseed pretreated with rumen fluid for 6 h (B) and 24 h (C).

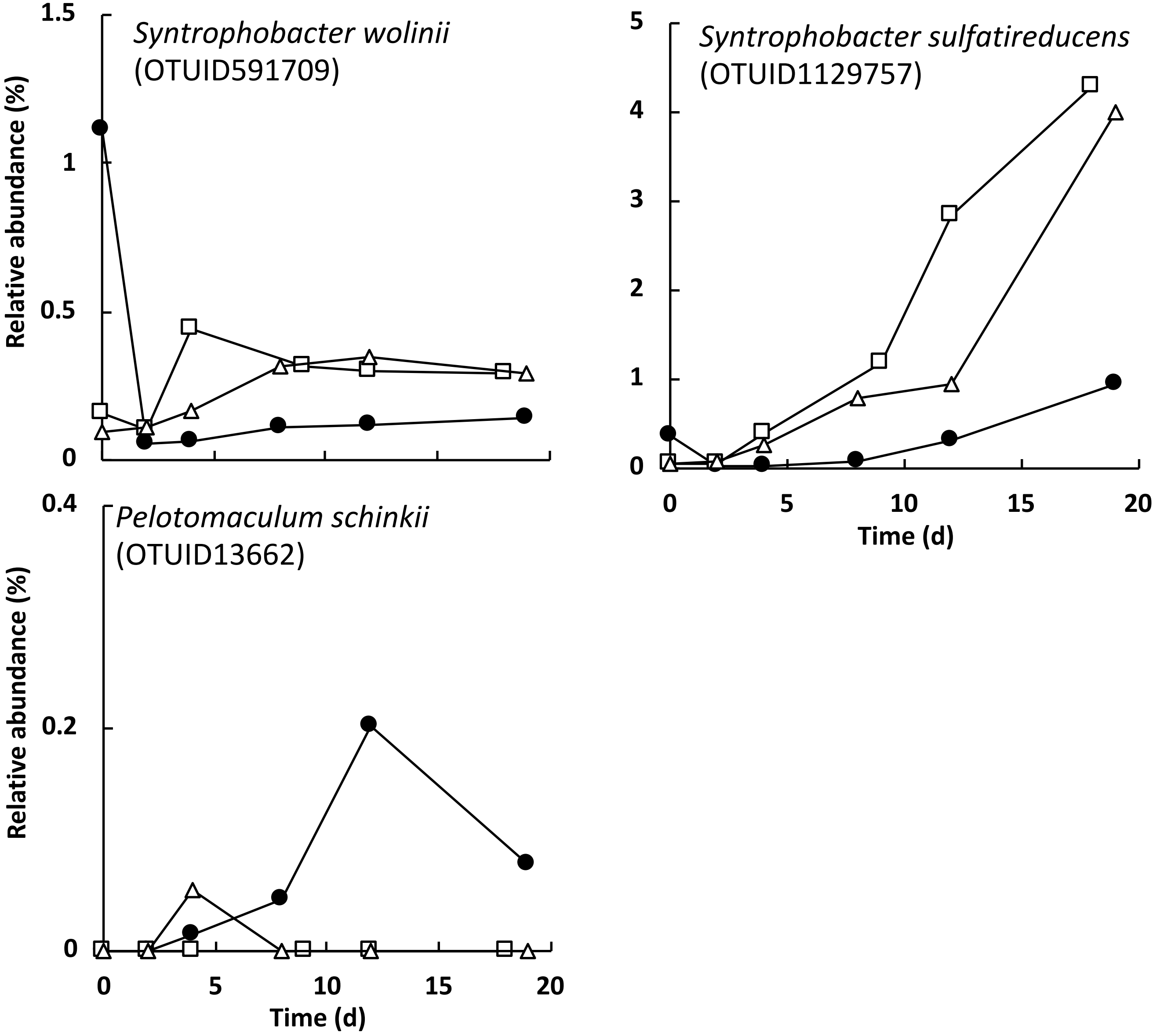

Fig. S10 (supplemental material)
Relative abundance of OTUs associated with known propionate-oxidizing bacteria during methane fermentation fed untreated rapeseed (●) and rapeseed pretreated with rumen fluid for 6 h (□) and 24 h (△).

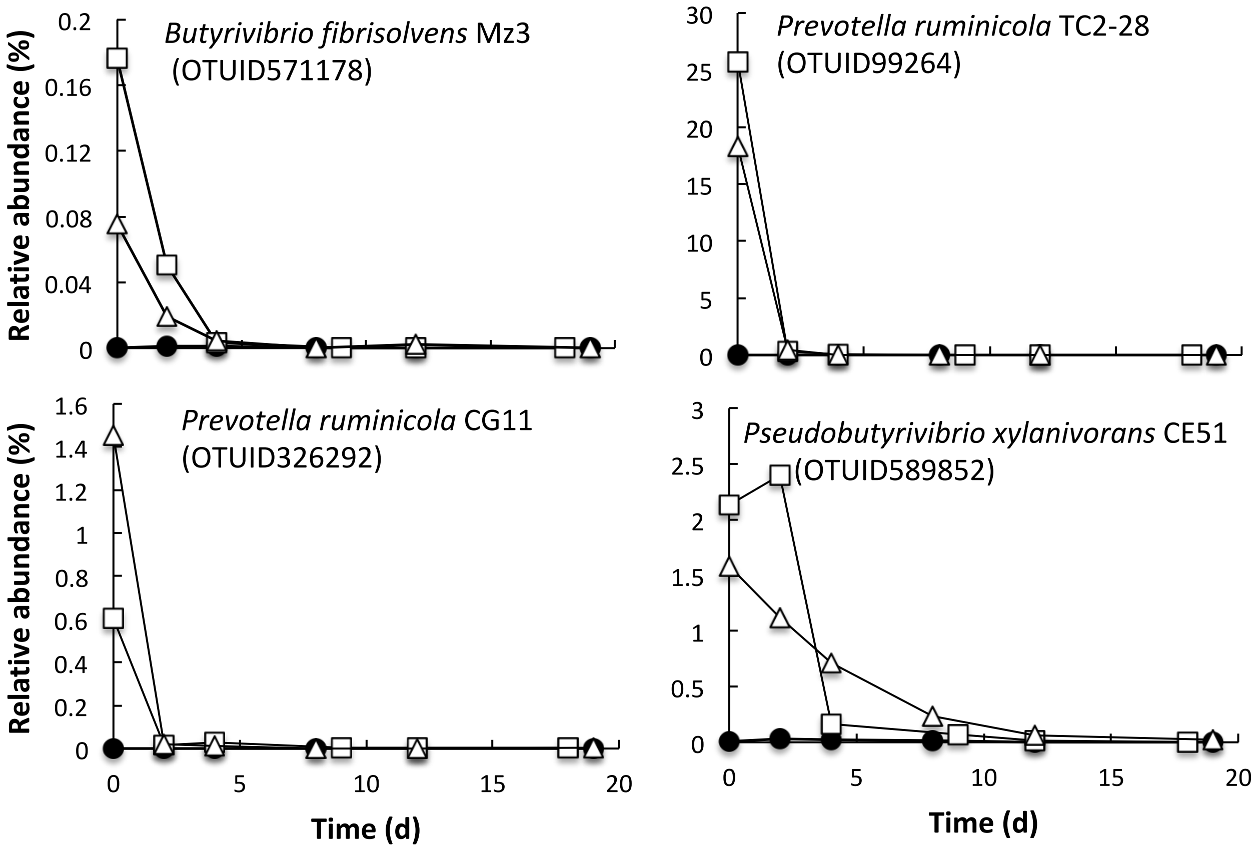

Fig. S11
Relative abundance of OTUs in Table S7 associated with known cellulolytic or xylanolytic rumen bacteria that were also detected in the untreated methane fermenter during methane fermentation fed untreated rapeseed (●) and rapeseed pretreated with rumen fluid for 6 h (□) and 24 h (△).

Table S1 Dissolved chemical oxygen demand (COD), volatile fatty acid (VFA) production, and pH after rumen fluid pretreatment for 6 and 24 h. [a]

	0 h	6 h	24 h
Dissolved COD (g/L)	7.95 ± 0.23	20.10 ± 0.57	23.60 ± 0.42
Volatile fatty acid production (g/L)			
Acetic acid (g/L)	2.79 ± 0.14	5.90 ± 0.24	8.10 ± 0.48
Propionic acid (g/L)	0.71 ± 0.05	2.03 ± 0.10	2.88 ± 0.20
Isobutyric acid (g/L)	0.04 ± 0.01	0.12 ± 0.02	0.11 ± 0.03
Butyric acid (g/L)	0.47 ± 0.08	0.84 ± 0.06	1.49 ± 0.05
Isovaleric acid (g/L)	0.09 ± 0.01	0.52 ± 0.61	0.13 ± 0.03
Valeric acid (g/L)	0.12 ± 0.16	0.09 ± 0.06	0.32 ± 0.02
pH	7.12 ± 0.26	6.09 ± 0.13	5.49 ± 0.04
Lignocellulose degradabilities (%)			
Cellulose	0	27.2	72.1
Hemicellulose	0	16.8	36.7
Lignin	0	16.9	22.5

[a] This table was made from the data in Baba et al. (2017) https://doi.org/10.1016/j.jbiosc.2016.11.008.

Table S2 *Synergistetes* taxa

Taxon	Most abundant OTU ID	Closest relative species of 16S rRNA gene	Accession No.	Identities
Aminobacterium	4396596	*Aminobacterium colombiense*	NR_074624	435/442(98%)
Thermovirgaceae	563237	*Thermovirga lienii* Cas60314	NR_043522	416/444(94%)
HA73	567883	*Aminivibrio pyruvatiphilus* 4F6E	NR_113331	436/442(99%)
vadinCA02	555847	*Clostridiales* bacterium JN18_A24_M	DQ168655	428/442(97%)

Table S3 *WWE1* taxa

Taxon	Most abundant OTU ID	Closest relative species of 16S rRNA gene	Accession No.	Identities
W22	539547	*Cloacimonetes* bacterium JGI 0000059-L07	KJ535434	457/467(98%)
W5	558528	Uncultured bacterium clone ATB_CK_926_35	KP150997	466/467(99%)

Table S4 *Bacteroidetes* taxa

Taxon (>1.0%)	Most abundant OTU ID *	Closest relative species of 16S rRNA gene	Accession No.	Identities
Bacteroides	572010 (C,P)	*Bacteroides graminisolvens*	NR_041642	459/460(99%)
Porphyromonadaceae	4312424 (C)	*Proteiniphilum acetatigenes*	MK414692	447/460(97%)
	541252 (P)	*Petrimonas mucosa* ING2-E5A	LT608328	459/460(99%)
Prevotella	768947 (C)	*Prevotella ruminicola* CG41	AB849451	459/460(99%)
	99264 (P)	*Prevotella ruminicola* TC2-28	AF218619	432/461(94%)
Bacteroidales	45529 (C)	Uncultured bacterium clone Ic118_27f	HQ259855	457/459(99%)
	574821 (P)	*Bacteroidales* bacterium P13	AB730729	449/460(98%)
Marinilabiaceae	544105 (C,P)	*Ruminofilibacter xylanolyticum* S1	DQ141183	458/460(99%)
RF16	100852 (C,P)	Rumen bacterium YS3	AF544208	455/459(99%)
S24-7	336446 (C,P)	Uncultured bacterium clone: Y14_2_39	KF007168	413/464(89%)
Paraprevotellaceae	143138 (C,P)	*Bacteroidales* bacterium R61	AB730657	459/460(99%)

* C, methane fermenter fed with untreated rapeseed (control); P, methane fermenter fed with pretreated rapeseed.

Table S5 Alpha diversity of bacterial communities in methane fermentation

	Control	6-h pretreatment	24-h pretreatment
shannon	5.980 ± 0.216	8.028 ± 0.272[†]	8.262 ± 0.276[†]
simpson	0.939 ± 0.015	0.975 ± 0.010[*]	0.982 ± 0.005[†]
ACE	2290 ± 125	4269 ± 283[†]	4278 ± 255[†]
chao1	2213 ± 119	4254 ± 269[†]	4224 ± 261[†]
goods_coverage	0.995 ± 0.001	0.992 ± 0.001	0.993 ± 0.001

[*] $P<0.05$, significantly different from the value of control (Mann–Whitney test)

[†] $P<0.01$, significantly different from the value of control (Mann–Whitney test)

TableS6 OTUs related to methanogenic archaea detected in methane seed sludge.

OTU ID	Relative species of 16S rRNA gene	Accession no.	Identity
706555	*Methanosarcina mazei* strain T40UCB9	MG008504	421/424(99%)
843826	*Methanospirillum stamsii* strain ps	NR_117705	412/419(98%)
826668	*Methanosaeta concilii* strain GP6	NR_102903	421/424(99%)
849920	Methanogenic archaeon DCM1	GQ339876	413/423(98%)
433016	*Methanoculleus chikugoensis*	KP702949	410/420(98%)
557417	*Methanospirillum* sp.Clone A2	AJ133792	409/420(97%)

Table S7 OTUs related to lignocellulose-degrading bacteria and methanogenic archaea detected in pretreatment fluid.[a]

OTU ID	Closest relative species of 16S rRNA gene	Annotation [b]	Accession No.	Identities
270433	*Ruminococcus albus* B199	C, X, O	AY445592	422/440(96%)
293178	*Ruminococcus flavefaciens* 17	C, X, O	AM748742	421/440(96%)
262032	*Fibrobacter succinogenes* R	C, X, O	AJ505938	456/458(99%)
571178	*Butyrivibrio fibrisolvens* Mz3	C, X, O	AM039822	438/441(99%)
14124	*Cellulosilyticum lentocellum* DSM 5427	C, X, O	NR_074536	440/442(99%)
99264	*Prevotella ruminicola* TC2-28	X, O	AF218619	432/461(94%)
326292	*Prevotella ruminicola* CG11	X, O	AB849444	426/460(93%)
589852	*Pseudobutyrivibrio xylanivorans* CE51	X, O	AM039824	439/440(99%)
554176	*Eubacterium uniforme* X3C39	X, O	NR_104842	438/440(99%)
572010	*Bacteroides graminisolvens* XDT-1	X, O	NR_041642	458/460(99%)
101860	*Lachnospira multipara* D32 (ATCC19207)	O	NR_104758	439/440(99%)
805261	*Succinivibrio dextrinosolvens* CA81	O	AB849337	427/440(97%)
539567	*Mesotoga infera* VNs100	O	NR_117646	464/467(99%)
303161	*Streptococcus equinus* HC5	O	KF573427	464/465(99%)
584375	*Bifidobacterium adolescentis* JCM 1275	O	LC071806	447/449(99%)
576472	*Bacteroides plebeius* M12	O	NR_041277	423/460(92%)
N[b]10999	*Prevotella copri* JCM 13464	O	NR_113411	423/460(92%)
4336330	*Xanthomonas sacchari* AF54	O	LC015609	461/465(99%)
659146	*Sedimentibacter hydroxybenzoicus* JW/Z-1	A	NR_029146	421/442(95%)
840291	*Acinetobacter indicus* A648	A	NR_117784	465/466(99%)
261405	*Soehngenia saccharolytica* DSM 12858	A	NR_117382	435/439(99%)
586744	*Brachymonas denitrificans* AS-P1	A	NR_025834	447/465(96%)
625556	*Syntrophorhabdus aromaticivorans* UI	A	NR_041306	440/469(94%)
842598	*Methanobrevibacter* sp. G16	M	KF697729	422/424(99%)
565174	*Methanosphaera stadtmanae* DSM 3091	M	CP000102	416/424(98%)
153647	*Methanosphaera* sp. ISO3-F5	M	KF697734	415/424(98%)
826668	*Methanosaeta concilii* GP6	M	NR_102903	421/424(99%)

[a] This table was made using the data in Baba et al. (2017) (https://doi.org/10.1016/j.jbiosc.2016.11.008).

[b] C, cellulose-degrading bacteria; X, xylan-degrading bacteria; A, aromatic-degrading bacteria; O, cello-oligosaccharide-degrading bacteria; M, methanogenic archaea

OTUs marked with gray color were not detected in methane seed sludge but were endemic to pretreatment (rumen) fluid.

Table S8 Propionate-oxidizing bacteria

OTU ID	Closest relative species of 16S rRNA gene	Accession No.	Identities
591709	*Syntrophobacter wolinii*	X70906	449/467(96%)
1129757	*Syntrophobacter sulfatireducens* TB8106	NR_043073	465/466(99%)
13662	*Pelotomaculum schinkii* HH	NR_119207	441/444(99%)
